# Artificial intelligence-aided assignment of journal submissions to associate editors—a feasibility study on IEEE transactions on medical imaging

**DOI:** 10.1186/s42492-025-00212-y

**Published:** 2026-01-12

**Authors:** Xuanang Xu, Joshua Yan, Gloria Nwachukwu, Hongming Shan, Uwe Kruger, Ge Wang

**Affiliations:** 1 Department of Biomedical Engineering and the Center for Biotechnology and Interdisciplinary Studies, Rensselaer Polytechnic Institute, Troy, NY 12180 United States; 2 Department of Radiology and Biomedical Imaging, Yale School of Medicine, New Haven, CT 06519 United States; 3 Shenendehowa High School, Clifton Park, NY 12065 United States; 4 Institute of Science and Technology for Brain-inspired Intelligence, Fudan University, Shanghai, 201203 China

**Keywords:** Associate editor assignment, Large language model, ModernBERT, Semantic similarity, Visualization

## Abstract

Efficient and accurate assignment of journal submissions to suitable associate editors (AEs) is critical in maintaining review quality and timeliness, particularly in high-volume, rapidly evolving fields such as medical imaging. This study investigates the feasibility of leveraging large language models for AE-paper matching in IEEE Transactions on Medical Imaging. An AE database was curated from historical AE assignments and AE-authored publications, and extracted six key textual components from each paper title, four categories of structured keywords, and abstracts. ModernBERT was employed locally to generate high-dimensional semantic embeddings, which were then reduced using principal component analysis (PCA) for efficient similarity computation. Keyword similarity, derived from structured domain-specific metadata, and textual similarity from ModernBERT embeddings were combined to rank the candidate AEs. Experiments on internal (historical assignments) and external (AE Publications) test sets showed that keyword similarity is the dominant contributor to matching performance. Contrarily, textual similarity offers complementary gains, particularly when PCA is applied. Ablation studies confirmed that structured keywords alone provide strong matching accuracy, with titles offering additional benefits and abstracts offering minimal improvements. The proposed approach offers a practical, interpretable, and scalable tool for editorial workflows, reduces manual workload, and supports high-quality peer reviews.

## Introduction

**Fig. 1:**
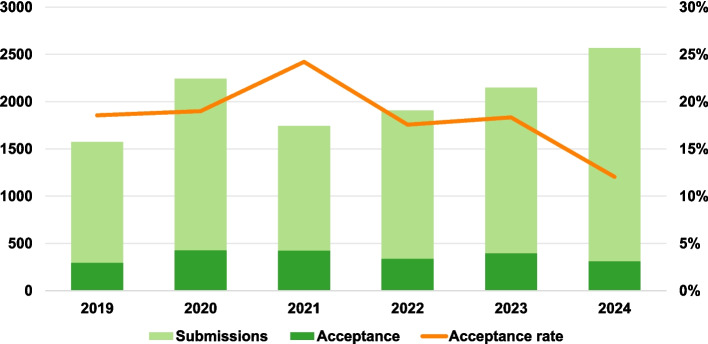
Annual statistics of IEEE TMI submissions and accepted papers from 2019 to 2024. Bars represent the number of original submissions and accepted papers. The orange curve indicates the acceptance ratio based on original submissions. Note that some papers submitted for 2024 are still pending peer review, which contributes to the lower acceptance rate compared to previous years

The management of academic journals, particularly in the rapidly evolving field of medical imaging, requires substantial editorial coordination, such as assigning submissions to suitable associate editors (AEs). IEEE Transactions on Medical Imaging (TMI), a leading journal in medical imaging, has been steadily growing in terms of the number of submissions, driven by the global rise in healthcare needs and associated research and development, particularly in AI (artificial intelligence)-powered research (Fig. 1). This has significantly increased the workload of managing editors responsible for manually matching each submission with a closely relevant AE. Inaccurate assignments lead to delayed review processes and compromised review quality, highlighting the need for a scalable and intelligent editorial workflow.

Large language models (LLMs), such as bidirectional encoder representations from transformers (BERT) [1] and ChatGPT [2], along with their variants, represent significant breakthroughs in natural language processing. Trained in extensive corpora, LLMs learn intricate language patterns and semantic representations, enabling them to effectively handle complex linguistic tasks. The advantages of LLMs include proficiency in capturing contextual nuances, high generalizability across diverse scenarios, and efficiency in automating tasks that traditionally require manual effort. Consequently, applying LLMs in processes, such as manuscript assignments, enhances accuracy and reduces the editorial workload.

Over the past few years, AI has made significant inroads into the workflows of journal management and peer review, including crucial components such as AE assignments. Frontier media introduced its AI review assistant in 2020, a tool designed to aid editors by identifying conflicts of interest, flagging plagiarism, assessing review quality, and recommending potential editors and reviewers. Meanwhile, AI-driven systems have streamlined other editorial tasks—such as automatic emailing, manuscript triage, and workflow automation—thereby reducing the administrative burden without replacing human oversight. In peer reviews, platforms such as AnnotateGPT leverage LLMs to generate manuscript annotations and highlights, thereby enhancing reviewer comprehension and efficiency. Simultaneously, studies evaluating AI integration into peer review underscore ethical and practical challenges, including limitations in evaluating novelty and domain expertise, concerns about bias and transparency, and the essentiality of human judgment in decision-making. For example, Liang et al. [3] conducted one of the first large-scale randomized controlled trials on the use of AI in top-tier computer science conference peer reviews, demonstrating that LLM-assisted reviewers produced more constructive, detailed, and higher-quality feedback than the control group, while highlighting the potential risks and the need for human oversight. These developments collectively illustrate the opportunities and challenges of AI in enhancing editorial workflows, particularly in sensitive areas such as AE assignments.

This study proposes a novel approach that leverages LLMs, specifically ModernBERT [4], to facilitate and automate the AE matching process for IEEE TMI. The proposed system employs a structured feature extraction methodology to parse critical text components from a given manuscript, followed by feature dimensionality reduction and similarity matching to associate submissions with suitable AEs. This study establishes the feasibility of using LLMs in this context, which is a key task in our AI for TMI initiative [5].

## Methods

### AE database curation

#### Data source

To establish an AI-based AE matching system, an AE database was created comprising:

- **Historical AE assignments:** A total of 1703 historical records were acquired from the IEEE TMI editorial office associated with 1703 papers handled by 129 unique AEs from June 2019 to December 2024. All the collected papers were accepted and published in TMI without any confidentiality or privacy issues. The published version was downloaded in PDF from IEEE Xplore. This collection represents the AEs’ experience in their job.
- **AE publications:** A total of 991 papers authored (including first-authored, co-authored, and corresponding-authored) by 105 AEs were collected using Google Scholar. These 105 AEs form a subset of the 129 AEs involved in the above “historical AE assignments” data, and were those who had established Google Scholar profiles. For each AE, 6–10 papers were selected, balancing between top-cited and most recent publications (approximately, half to half). The final published version (or preprint version if not published) was downloaded in PDF from the publishers’ official website (or preprint platform such as arXiv and medRxiv). The selected papers represent the AEs’ expertise in the past and up to date.

#### Data split

The “historical AE assignments” data was split by dividing the papers handled by each AE into two parts: subset 1 (80%), a total of 1362 historically assigned papers involving 129 distinct AEs. Subset 2 (20%): 341 historically assigned studies involving 120 distinct AEs. All 120 AEs in Subset 2 were also included in Subset 1, but Subset 1 included nine additional AEs that had only one assigned paper. Subset 1 from the “historical AE assignments” data was used as the training set. For evaluation, Subset 2 from the “historical AE assignments” data was used as the internal testing set, where the developed model was tested on unseen papers following the same data distribution as the training set. Furthermore, the “AE Publications” data was used as the external testing set and assumed a scenario that the AEs submit their papers to TMI for review. In this case, the paper should match their expertise most closely because they know the paper better than anyone else.

#### Data preprocessing

Given a TMI submission, its content can be summarized by title, keywords, and abstract. Four types of keywords were defined to describe the given submissions as follows:

- **Keywords regarding imaging modalities:** These keywords categorize the paper by the medical imaging modalities concerned in the study. Twenty-two candidate keywords were provided for the specification of the imaging modalities: ‘Angiographic imaging’, ‘Diffusion weighted imaging’, ‘Electrical Impedance Tomography’, ‘Electroencephalogram’, ‘Electrophysical imaging’, ‘Endoscopy’, ‘Fluorescence tomography’, ‘Functional imaging’, ‘Magnetic Particle Imaging’, ‘Magnetic resonance imaging’, ‘Microscopy’, ‘Microwave’, ‘Molecular and cellular imaging’, ‘Nuclear imaging’, ‘Optical Imaging/OCT/DOT’, ‘Optoacoustic/photoacoustic imaging’, ‘Others’, ‘Perfusion imaging’, ‘Thermal imaging’, ‘Ultrasound’, ‘Viscoelasticity imaging’, ‘X-ray imaging and computed tomography’.
- **Keywords regarding imaging targets:** These keywords categorize the paper by the objects of interest involved in the study. Thirty-six candidate keywords were provided for the choice of the objects of interest: ‘Abdomen’, ‘Animal models and imaging’, ‘Arm and Hand’, ‘Bone’, ‘Brain’, ‘Breast’, ‘Cell’, ‘Cervix’, ‘Eye’, ‘Fetus’, ‘Gastrointestinal tract’, ‘Genome’, ‘Head and neck’, ‘Heart’, ‘Inner ear’, ‘Kidney’, ‘Lesion and tumor’, ‘Liver’, ‘Lung’, ‘Lymphatic system’, ‘Muscle’, ‘Nerves’, ‘Neurological disorders’, ‘Organs’, ‘Others’, ‘Pediatric and adolescent’, ‘Pelvis’, ‘Prostate’, ‘Skin’, ‘Spine’, ‘Thorax’, ‘Thyroid’, ‘Tissue’, ‘Tooth’, ‘Vessels’, ‘Whole-body’.
- **Keywords regarding general methodologies:** These keywords categorize the paper by the methodologies used or studied. One hundred and seventeen candidate keywords were provided on methodologies: ‘3D reconstruction’, ‘Anatomical priors’, ‘Artificial intelligence’, ‘Atlases’, ‘Attention mechanism’, ‘Biomechanical modeling’, ‘Blind source separation’, ‘CT physics’, ‘Collaborative Learning’, ‘Compressed sensing’, ‘Compressive sensing’, ‘Computational geometry’, ‘Computer-aided detection and diagnosis’, ‘Connectivity analysis’, ‘Continual learning’, ‘Data Mining’, ‘Dataset’, ‘Deep learning’, ‘Digital twins’, ‘Dimensionality reduction’, ‘Domain adaptation’, ‘Domain generalization’, ‘End-to-end learning in medical imaging’, ‘Evaluation and Performance’, ‘Federated Learning’, ‘Few-shot learning’, ‘Finite element modeling’, ‘Foundation model’, ‘Generative modeling’, ‘Geometric deep learning’, ‘Graph network models’, ‘Graph theory’, ‘Human-in-the-loop’, ‘Image acquisition’, ‘Image analysis’, ‘Image enhancement/restoration’, ‘Image preprocessing’, ‘Image processing’, ‘Image quality assessment’, ‘Image reconstruction’, ‘Image registration’, ‘Image synthesis’, ‘Image-guided treatment’, ‘Imaging’, ‘Imaging Modality’, ‘Information theory’, ‘Integration of multiscale information’, ‘Interpretability’, ‘Inverse methods’, ‘Longitudinal modeling’, ‘Loss function’, ‘Low-rank modeling’, ‘MR physics’, ‘Machine learning’, ‘Manifold data’, ‘Masked AutoEncoder’, ‘Mathematical modeling’, ‘Medical robotics’, ‘Microwave’, ‘Model-based methods’, ‘Motion compensation and analysis’, ‘Multi-modality fusion’, ‘Multiple Instance Learning’, ‘Multitask learning’, ‘Multiview learning’, ‘Natural language processing’, ‘Neural network’, ‘Object detection’, ‘Optimization’, ‘Others’, ‘Parallel computing’, ‘Pattern recognition and classification’, ‘Personalization’, ‘Personalized healthcare’, ‘Phase contrast CT’, ‘Physics-informed neural networks’, ‘Population analysis’, ‘Privacy preservation’, ‘Probabilistic and statistical methods’, ‘Prognosis and prediction’, ‘Quantification and estimation’, ‘ROC analysis’, ‘Radiomics’, ‘Recurrent neural networks’, ‘Registration’, ‘Reinforcement learning’, ‘Representation learning’, ‘Robust learning’, ‘Score-based Generative Models’, ‘Segmentation’, ‘Self-supervised learning’, ‘Semi-supervised learning’, ‘Shape analysis’, ‘Signal processing’, ‘Sparse modelling’, ‘Spatio-temporal modeling’, ‘Statistical features’, ‘Stereoscopy’, ‘Structure-function coupling’, ‘Super resolution’, ‘Surgical guidance/navigation’, ‘System design’, ‘Tissue modelling’, ‘Topology’, ‘Tracking’, ‘Tractography’, ‘Transformer models’, ‘Ultrasound’, ‘Uncertainty estimation’, ‘Unsupervised learning’, ‘Validation’, ‘Variational method’, ‘Virtual/augmented reality’, ‘Visualization’, ‘Weakly-supervised learning’, ‘X-ray imaging and computed tomography’, ‘fMRI analysis’.
- **Keywords defined by the authors:** These keywords were defined by the authors and listed after the abstract in the submission. It could be generally more accurate and comprehensive to summarize the paper’s topic, but more flexible and diverse.

Combined with the paper title and abstract, The following six parts of textual information were utilized to characterize the given submission:

1. Title
2. Keywords (Imaging Modalities)
3. Keywords (Object of Interest)
4. Keywords (General Methodologies)
5. Keywords (Author Defined)
6. Abstract

Because the papers collected in the AE database were all in PDF form from different publishers with diverse formats, mixed with figures and tables, the raw files were preprocessed, and the aforementioned six pieces of information were extracted. This was achieved using ChatGPT (GPT-4o) [2] with a specifically designed prompt. Owing to ChatGPT’s throughput limitations, 3–5 PDF files were processed each time, and a new chat was restarted using the same prompt for each session. The prompts were as follows:

Please extract the abstract from the attached papers. Do not make any changes to the contents of the original abstract.

Please select at least two keywords that are mostly associated with the attached paper from each of the three lists of keyword categories:

(1) Imaging Modalities: Angiographic imaging, Bioluminescence tomography, Diffusion weighted imaging, Electrical Impedance Tomography, Electroencephalogram (EEG), Electrophysical imaging, Endoscopy, Fluorescence tomography, Functional imaging (e.g., fMRI), Magnetic Particle Imaging, Magnetic resonance imaging (MRI), Microscopy, Microwave, Molecular and cellular imaging, Nuclear imaging (e.g., PET SPECT), Optical Imaging/OCT/DOT, Optoacoustic/photoacoustic imaging, Perfusion imaging, Thermal imaging, Ultrasound, Viscoelasticity imaging, X-ray imaging and computed tomography, Others;

(2) Object of Interest: Abdomen, Animal models and imaging, Bone, Brain, Breast, Cell, Cervix, Eye, Fetus, Gastrointestinal tract, Genome, Heart, Inner ear, Kidney, Liver, Lung, Muscle, Nerves, Prostate, Skin, Spine, Thyroid, Tooth, Vessels, Others;

(3) General Methodologies: Active imaging, Atlases, Biomechanical modeling, Blind source separation, Collaborative Learning, Compressive sensing, Computational geometry, Computer-aided detection and diagnosis, Connectivity analysis, CT physics, Data Mining, Decentralized Learning, Dimensionality reduction, End-to-end learning in medical imaging, Evaluation and Performance, Federated Learning, Finite element modeling, fMRI analysis, Foundation model, Geometric deep learning, Graph network models, Graph theory, Image acquisition, Image enhancement/restoration (noise and artifact reduction), Image-guided treatment, Image quality assessment, Image reconstruction, Integration of multiscale information, Inverse methods, Machine learning, Manifold data, Medical robotics, Motion compensation and analysis, MR physics, Multi-modality fusion, Natural language processing, Neural network, Optimization, Parallel computing, Pattern recognition and classification, Phase contrast CT, Population analysis, Probabilistic and statistical methods, Quantification and estimation, Radiomics, Registration, ROC analysis, Score-based Generative Models, Segmentation, Shape analysis, Stereoscopy, Super resolution, Surgical guidance/navigation, System design, Tissue modelling, Tracking (time series analysis), Tractography, Validation, Virtual/augmented reality, Visualization, Others.

The output format should be “Filename: [Filename of the attached paper]\nTitle: [Title]\nKeywords (Imaging Modalities): [keywords selected from “Imaging Modalities” list separated by “,”]\nKeywords (Object of Interest): [keywords selected from “Object of Interest” list separated by “,”]\nKeywords (General Methodologies): [keywords selected from “General Methodologies” list separated by “,”]\nKeywords (Author Defined): [keywords provided in the attached paper or, if missing, 3–5 keywords summarized according to the paper’s contents (all keywords should be separated by “,”)]\nAbstract: [Abstract contents]” Please reformat the abstract in a single paragraph. Do not start a new line between “Abstract” and the main text of the abstract. No need to put a link to the source paper in the output.

If there are multiple attached papers, repeat the above process for each of them and sequentially output the results. No need to number the papers.

Notably, the above preprocessing procedure was only required during our AE database curation because these papers were collected from diverse sources and organized in different formats. Given any TMI submission, when the authors submitted their paper to TMI in the submission system, they were asked to manually type or select the above six parts of the information. Therefore, textual information can be directly obtained from the IEEE Xplore system.

**Fig. 2:**
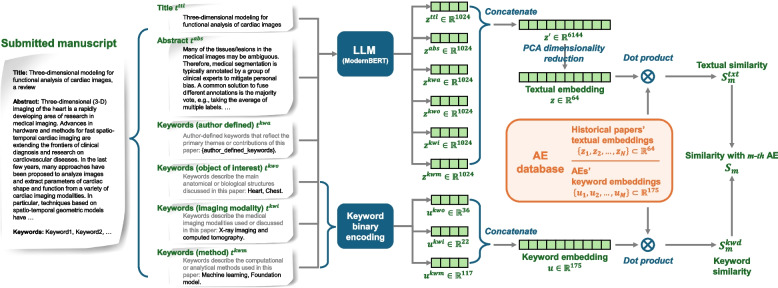
Scheme of the proposed AE matching approach

### Method design

The overall workflow of the proposed AE matching framework is depicted in Fig. 2. For each newly submitted manuscript, the content was first decomposed into multiple textual components. These components were then processed using two complementary encoders: an LLM to generate semantic textual embeddings and a keyword encoder to produce structured keyword embeddings. The resulting feature representations were compared with the corresponding embeddings stored in the AE database, and the most relevant AEs were identified based on their similarity scores.

#### Textual similarity

**Textual feature extraction:** To quantify the similarity/dissimilarity between any pair of profiles in terms of the above six semantic features, LLM was used to convert textual information to semantic embeddings. Considering that our approach was used to process submitted papers that are not yet publicly available, the use of closed-source or cloud-based LLMs should be avoided to protect the confidentiality and privacy of the submissions. Therefore, ModernBERT [4] was used to process the submission locally, because it can be deployed in local environments with limited resources within firewalls. ModernBERT is an advanced encoder-only transformer model specifically designed to address several key limitations of the original BERT model and its successors. It was introduced by Answer.AI and LightOn in December 2024. ModernBERT exhibits faster speed and higher accuracy across a range of tasks than its precursors, such as BERT [1] and RoBERTa [6]. It supports a sequence length of 8192 tokens, which is a significant increase over BERT’s 512 tokens. This allows for effective and efficient handling of longer documents and more complex data.

The six extracted prompts $\left\{t^{ttl}, t^{kwo}, t^{kwi}, t^{kwm}, t^{kwa}, t^{abs}\right\}$ were fed into ModernBERT to convert them into a latent embedding, which is the class token (i.e., the first token of the output sequence) to serve as the feature representation of the corresponding prompt. The title and abstract were directly fed into this LLM because they were already well organized in terms of grammar and semantic meaning. Because the keywords are individual English words without context, they were reformatted using the following template to make them readable before feeding them into the LLM:

- $$t^{ttl}$$ = “Paper title”
- $t^{kwo}$ = “The following keywords describe the main anatomical or biological structures discussed in this paper: Object of interest keyword list.”
- $t^{kwi}$ = “The following keywords describe the medical imaging modalities used or discussed in this paper: imaging modality keyword list.”
- $t^{kwm}$ = “The following keywords describe the computational or analytical methods used in this paper: methodology keyword list.”
- $t^{kwa}$ = “The following are author-defined keywords that reflect the primary themes or contributions of this paper: author defined keyword list.”
- $$t^{abs}$$ = “Paper abstract”

Each of the six extracted components was processed individually using ModernBERT to generate a 1024-dimensional feature vector (ModernBERT-large model’s class token has a dimension of 1024). Concatenating these six vectors $\left\{z^{ttl}, z^{kwo}, z^{kwi}, z^{kwm}, z^{kwa}, z^{abs}\right\}\subset {\textbf {R}}^{1024\times 1}$ yields a combined 6144-dimensional representation ${\textbf {z}}'\in {\textbf {R}}^{6144\times 1}$ for each paper, which summarizes the submission.

**Feature dimensionality reduction:** To enhance computational efficiency without significantly compromising discriminative power, principal component analysis (PCA) was applied to reduce the dimensionality of feature vectors from the original high dimensionality $D=6144$ to a low dimensionality $D=64$. The projection matrix ${\textbf {M}}_{PCA}\in {\textbf {R}}^{64\times 6144}$ of PCA was calculated according to the representation features extracted from papers in the training set. In this study, six parts of textual information $\left\{t^{ttl}, t^{kwo}, t^{kwi}, t^{kwm}, t^{kwa}, t^{abs}\right\}$ were extracted first, and the corresponding representation feature ${\textbf {z}}'\in {\textbf {R}}^{6144\times 1}$ was then constructed using ModernBERT and then reduced dimensionality using PCA:

1 $$\begin{aligned} {\textbf {z}}={\textbf {M}}_{PCA}^{64\times 6144}\times {\textbf {z}}'\in {\textbf {R}}^{64\times 1} \end{aligned}$$

This produced an optimized compact representation for the given study.

**Textual similarity calculation:** For *N* papers $\{X_i\}_{i=1}^{N}$ in the training set of the AE database associated with the individual AEs $\{Y_{i}|Y_{i}\in \{1,2,\ldots ,M\}\}_{i=1}^{N}$ were mapped to the feature space $\{{\textbf {z}}_1,{\textbf {z}}_2,\ldots ,{\textbf {z}}_N\}$ using the above feature extraction and dimensionality reduction methods. Given an unseen test paper $X_{test}$ from the internal testing set or the external testing set in Data split subsection, the same extraction and compression procedures were applied to obtain a corresponding feature vector ${\textbf {z}}_{test}$. The following similarity metric was then computed between this feature vector ${\textbf {z}}_{test}$ and all feature vectors for the AEs $\{{\textbf {z}}_1,{\textbf {z}}_2,\ldots ,{\textbf {z}}_N\}$:

2 $$\begin{aligned} s_i={CosSim}({\textbf {z}}_{test},{\textbf {z}}_i),\quad i=1,2,\ldots ,N \end{aligned}$$

where $CosSim(\cdot )$ represents the cosine similarity function. The similarity values were then grouped by the associated AE labels and aggregated the similarity values in the same group to yield a textual similarity value for the *m*th AE:

3 $$\begin{aligned} S_m^{txt}=Aggr(\{s_i\}),\quad \forall \ Y_i=m \end{aligned}$$

where $Aggr(\cdot )$ represents the aggregation function. The maximum function was employed for the aggregation.

#### Keyword similarity

**AE keyword feature extraction:** For the three enumerate type of keywords (i.e., the keywords for imaging modality, object of interest, and methodology) of the *N* papers $\left\{[t_i^{kwo},t_i^{kwi},t_i^{kwm}]\right\}_{i=1}^N$ contained in the training set from the AE database were mapped to three one-hot vectors $\left\{[{\textbf {u}}_i^{kwo},{\textbf {u}}_i^{kwi},{\textbf {u}}_i^{kwm}]\right\}_{i=1}^N$. For example, a 22-dimensional one-hot (binary) vector representing the keywords for imaging modalities selected from the 22 candidate keywords was defined. If a candidate keyword is selected for the paper, the corresponding bit will be set to 1; otherwise, 0. Similarly, the keywords for the objects of interest and methodology can be converted into 36-dimensional and 117-dimensional one-hot vectors, respectively. By concatenating the three one-hot vectors, keywords for each paper can be represented by a 175-dimensional one-hot vector ${\textbf {u}}_i\in \{0,1\}^{175\times 1}$. For the m-th AE in the database, the (normalized) averaged keyword vector ${\textbf {U}}_m$ was calculated to represent the AE’s relative strength of expertise.

4 $$\begin{aligned} {\textbf {U}}_m=\frac{mean(\{{\textbf {u}}_i\})}{\Vert mean(\{{\textbf {u}}_i\})\Vert },\forall \ Y_i=m \end{aligned}$$

**Keyword similarity calculation:** Given an unseen paper $X_{test}$ submitted to TMI, the above process was employed to extract its 175-dimensional keyword feature vector ${\textbf {u}}_{test}\in {0,1}^{175\times 1}$. The cosine similarity between this paper’s keyword feature vector ${\textbf {u}}_{test}$ and each AE’s keyword vector ${\textbf {U}}_m$ was then calculated to yield the keyword similarity value:

5 $$\begin{aligned} S_{m}^{kwd}=CosSim({\textbf {u}}_{test}, {\textbf {U}}_m),\quad m=1,2,\ldots ,M \end{aligned}$$

It is worth noting that the textual similarity and keyword similarity use different aggregation strategies, as they capture complementary types of information. For textual similarity, the maximum similarity between the test paper and all papers handled by an AE was considered. Because the AE may have broad research experience across diverse topics, the maximum operator identifies the closest match and prevents highly relevant experiences from being diluted by fewer related papers. This design aligns with actual editorial practice in which an AE remains highly suitable as long as they have strong expertise in submission. Conversely, keyword similarity was designed to represent an AE’s overall expertise profile. Therefore, the normalized average of the keyword vectors across all papers handled by each AE was computed to produce a stable and noise-reduced representation of their overall expertise. These two aggregation mechanisms complement each other by jointly capturing local (paper-level) similarity and global (profile-level) expertise.

#### AE similarity

The overall similarity between a submitted paper $X_{test}$ and the *m*th AE in the AE database is defined as the sum of the textual and keyword similarities:

6 $$\begin{aligned} S_m=S_m^{txt}+S_m^{kwd},\quad m=1,2,\ldots ,M \end{aligned}$$

By calculating such AE similarity values over all AEs in the AE database, the affinity between the submission $X_{test}$ and all the AEs in the database could be quantified. A higher value of this AE similarity indicates that the AE is more suitable for handling the submission.

#### Evaluation metrics

To evaluate the performance of the proposed approach, two metrics were employed: the top-K accuracy and top-K similarity.

**Top-K accuracy:** Given a testing paper $X_{test}$ and the ground-truth AE label $Y_{test}$ from the testing set of the AE database, its similarity values $\{S_m\}_{m=1}^M$ with all AEs in the database were calculated. If the ground-truth AE label $Y_{test}$ appeared in the top K highest similarity values, the result was counted as a correct match. Otherwise, the result is counted as an incorrect match. The percentage of correct matching over all the testing papers in the testing set was the top-K accuracy.

7 $$\begin{aligned} Acc_{topK}=\frac{\#\ of\ correct\ matching}{\#\ of\ total\ matching} \end{aligned}$$

The top-5, top-10, top-30, and top-50 accuracy were calculated to comprehensively assess the approach.

**Top-K similarity:** In addition to accuracy, the top-K similarity was introduced as a complementary metric to quantify how semantically close the top-K predicted AEs are to the ground-truth AE, regardless of whether the exact AE label is present in the top-K list. Specifically, for each testing paper, the cosine similarity between the ground-truth AE’s average keyword vector and each of the top-K ranked AEs’ vectors was calculated, and their average was computed. The top-K similarity is defined as follows:

8 $$\begin{aligned} Sim_{topK}=\frac{1}{K} \sum \limits _{k=1}^K CosSim\left({\textbf {U}}_{Y_{test}} ,{\textbf {U}}_{\hat{Y}_k}\right) \end{aligned}$$

where ${\textbf {U}}_{Y_{test}}$ is the keyword vector of the ground-truth AE and $\{{\textbf {U}}_{\hat{Y}_k}\}_{k=1}^K$ are the keyword vectors of the top-K predicted AEs. This metric captures the semantic proximity between the correct AE and the system’s top K candidate AEs, thereby providing a continuous measure of fitness instead of a binary correctness.

## Results

### Data analysis on AE database

**Fig. 3:**
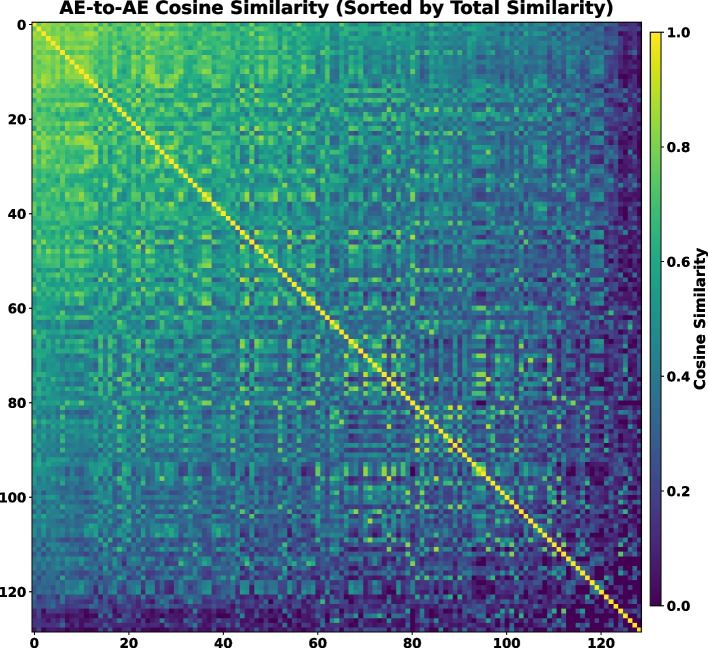
Cosine similarity matrix of 129 AEs’ average keyword vectors in the training set. Each row/column represents an AE, with the number of papers they handled indicated in parentheses. AEs are sorted in descending order by their total similarity to others; therefore, those positioned toward the top-left exhibit broader similarity across the group. Color intensity reflects the degree of similarity, with brighter values indicating more similar AE expertise profiles

In Keyword similarity subsection, the average keyword vector ${\textbf {U}}$ was defined to represent each AE’s expertise computed from the one-hot embeddings of three enumerated types of keywords—namely, imaging modality, object of interest, and methodology—extracted from the papers they handled. Based on these vectors, the cosine similarity between each pair of AEs was calculated to quantify the semantic closeness of their expert profiles. Figure 3 presents the resultant similarity matrix, where each row or column corresponds to an AE and the color intensity reflects the degree of similarity between their average keyword vectors. Brighter entries indicate stronger similarities. The AEs were sorted in descending order based on their total similarity with all the others. Consequently, those positioned at the top left of the matrix exhibited broader or more overlapping expertise with their peers. This provides an interpretable global view of the distribution of editorial expertise. This is an interesting utility of the averaged keyword vectors in capturing expertise overlaps among AEs.

**Fig. 4:**
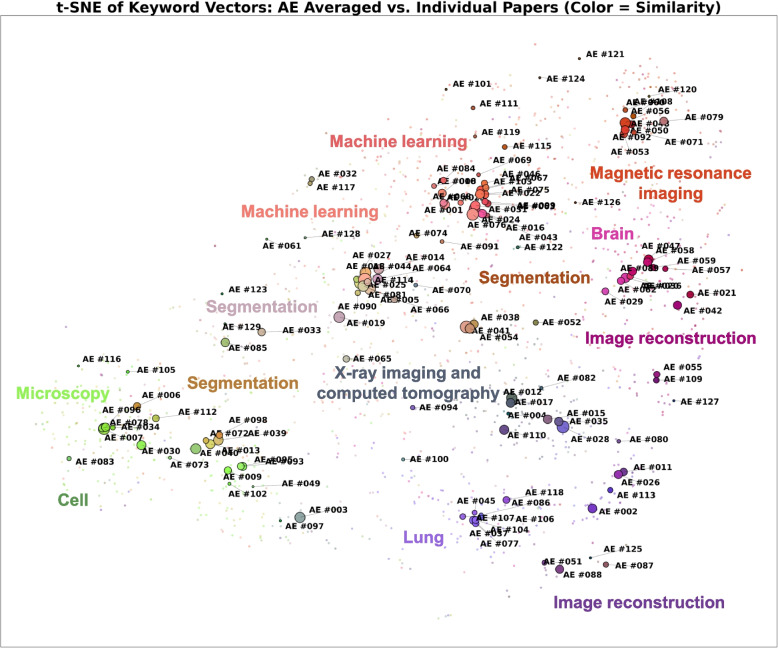
*t*-SNE visualization of all papers’ keyword vectors (small points) and AEs’ averaged keyword vectors (large points) from the training set. Colors are based on the similarity of AEs’ averaged keyword vectors; hence, similar colors indicate similar keyword distributions. The size of each large point is proportional to the number of papers handled by the corresponding AE

To visualize the coverage of AEs’ expertise in a low-dimensional space, *t*-distributed stochastic neighbor embedding (*t*-SNE) [7] was applied to these keyword vectors. Specifically, both the individual paper-level keyword vectors and the AEs’ averaged keyword vectors were projected into a shared 2D space. As shown in Fig. 4, small points represent the keyword vectors of individual papers, whereas large points correspond to the AEs’ average keyword vectors from the training set. To enhance interpretability, colors were assigned based on the similarity between AEs’ average keyword vectors, such that AEs with more similar expertise profiles were rendered in similar colors. This visualization complements the similarity matrix, offering an intuitive understanding of the distribution and clustering of editorial expertise across the entire database.

### Effectiveness of key components

To assess the individual and combined contributions of the key components in the proposed AE matching framework, an ablation study was conducted on both the internal testing set (historical AE assignments) and the external testing set (AE Publications). The performance was evaluated using both the top-K accuracy and top-K similarity metrics for K = 5, 10, 30, and 50, as summarized in Tables 1 and 2, respectively.

**Table 1:** Ablation study on the internal testing set (historical AE assignments) evaluating the individual and combined contributions of textual similarity, keyword similarity, and PCA enhancement

Model	PCA	Top-K accuracy ($\%$)				Top-K similarity			
		K = 5	K = 10	K = 30	K = 50	K = 5	K = 10	K = 30	K = 50
Textual similarity only	✗	09.68	20.82	47.21	65.10	0.5646	0.5547	0.5303	0.5153
	✓	11.14	20.53	47.21	66.86	0.5581	0.5514	0.5298	0.5161
Keyword similarity only	N/A	36.66	51.03	78.89	**88.56**	0.6677	0.6479	0.5955	0.5641
Textual + Keyword similarity	✗	**36.95**	50.73	78.59	**88.56**	0.6679	0.6484	0.5960	0.5646
	✓	36.36	**53.67**	**80.35**	85.04	**0.6800**	**0.6579**	**0.6007**	**0.5673**

Metrics include top-K accuracy and top-K similarity for K = 5, 10, 30, and 50, respectively. Keyword similarity alone gives strong performance, and combining it with textual similarity yields better results. PCA improves textual features by reducing dimensional noise

**Table 2:** Evaluation results on the external testing set (AE Publications) using the same ablation protocol

Model	PCA	Top-K accuracy ($\%$)				Top-K similarity			
		K = 5	K = 10	K = 30	K = 50	K = 5	K = 10	K = 30	K = 50
Textual similarity only	✗	09.14	12.96	34.05	51.00	0.5013	0.4926	0.4816	0.4714
	✓	08.97	14.12	34.72	49.34	0.4934	0.4877	0.4779	0.4692
Keyword similarity only	N/A	**26.08**	36.71	**56.31**	**70.60**	0.5887	0.5725	0.5382	0.5155
Textual + Keyword similarity	✗	25.75	36.54	55.98	**70.60**	0.5896	0.5733	0.5388	0.5159
	✓	25.91	**37.04**	55.65	67.28	**0.5977**	**0.5812**	**0.5422**	**0.5161**

Performance trends are consistent with those of internal testing: keyword similarity plays a central role, and integrating it with PCA-enhanced textual features yields the best performance across metrics

Across both datasets, keyword similarity consistently outperformed textual similarity when used in isolation, underscoring the strength of structured domain-specific keyword matching for capturing AEs’ expertise. For example, in the internal testing set, keyword similarity alone achieved a top-5 accuracy of 36.66%, compared with 9.68% with textual similarity. When both components were combined, further gains were evident, particularly when PCA was applied to refine textual embeddings by reducing dimensional noise. In the internal testing set, the full model with PCA achieved 36.36% top-5 accuracy of 53.67% top-10 accuracy, and 80.35% top-30 accuracy, representing the best overall performance. The external testing set showed the same trend, although with lower absolute scores owing to domain shift and topical diversity. Keyword similarity alone remained strong (26.08% top-5 accuracy), whereas textual similarity alone was considerably weaker (9.14% top-5 accuracy). Moreover, the best results were achieved when both similarity components were combined with PCA-enhanced textual features.

These results validate that keyword similarity is the primary driver of AE-paper matching performance, while textual similarity plays a complementary but significant role—its contribution becomes most effective when combined with keywords and optimized through PCA.

### Ablation study on textual information

To further evaluate the contribution of individual textual components to the AE matching performance, an ablation study was conducted by selectively including or excluding the title, four types of keywords (imaging modalities, object of interest, methodologies, and author-defined keywords), and the abstract during feature extraction. The results are summarized in Table 3.

**Table 3:** Ablation study on the internal testing set evaluating different combinations of textual components—title, four types of structured keywords, and abstract—for AE matching

Model						Top-K accuracy ($\%$)				Top-K similarity			
Title	Keyword				Abstract	K = 5	K = 10	K = 30	K = 50	K = 5	K = 10	K = 30	K = 50
	Modality	Object	Method	Author									
✓	✗	✗	✗	✗	✗	36.07	**53.67**	79.47	86.22	0.6769	0.6584	0.6005	0.5663
✗	✓	✓	✓	✓	✗	36.95	51.32	**81.23**	88.86	0.6773	0.6533	0.6002	0.5671
✗	✗	✗	✗	✗	✓	34.31	52.20	79.77	**89.15**	0.6783	0.6573	**0.6029**	**0.5697**
✓	✓	✓	✓	✓	✗	**37.83**	53.37	78.89	86.80	0.6784	0.6576	0.6004	0.5668
✗	✓	✓	✓	✓	✓	37.24	51.61	79.77	88.86	0.6793	0.6568	0.6012	0.5688
✓	✗	✗	✗	✗	✓	36.95	53.37	78.89	85.34	0.6799	**0.6585**	0.6008	0.5668
✓	✓	✓	✓	✓	✓	36.36	**53.67**	80.35	85.04	**0.6800**	0.6579	0.6007	0.5673

Performance differences across various configurations are relatively small, indicating that textual similarity plays a secondary role compared to keyword similarity. Among single-source inputs, keywords yielded the highest accuracy, followed closely by title, while the abstract alone was less effective. Combining the title and keywords provides modest gains, but adding the abstract offers no major improvement

Overall, the performance differences between the various textual combinations were relatively small, with top-5 accuracy values ranging narrowly from 34.31% to 37.83%. This limited variation aligns with earlier findings in Effectiveness of key components subsection (Tables 1 and 2) that textual similarity plays a supportive but nondominant role in AE matching, contributing to a modest performance gain compared to the much stronger impact of keyword similarity. Among these components, using only four types of keywords achieved the highest performance (36.95% top-5 accuracy), confirming that structured keywords capture substantial domain-specific signals for AE matching. Only the title yielded a slightly lower but still competitive performance (36.07%), reflecting the title’s importance in succinctly conveying the manuscript’s theme. However, only the abstract produced a lower accuracy (34.31%), indicating that abstracts, while more verbose, may include broader contextual or background information that is less discriminative for AE expertise matching. When the title was combined with all four types of keywords, the accuracy increased slightly to 37.83%, indicating that these two elements provided complementary signals. However, adding an abstract to this combination did not yield further improvements, suggesting redundancy in the abstract content with the title and keywords.

These results suggest that while textual features offer supplementary information, keyword matching remains the dominant factor in AE-paper affinity modeling in our framework. The relatively small differences between the textual configurations indicate that the model performance is robust to the specific choice of textual inputs as long as structured keyword features are included.

## Discussion

In the proposed approach, cosine similarity was chosen to compare feature vectors, although a negative Euclidean distance could theoretically serve a similar purpose. This decision was motivated by two considerations: First, ModernBERT embeddings primarily encode semantic information in terms of the direction in the embedding space, whereas the Euclidean distance is sensitive to the vector magnitude. Cosine similarity emphasizes angular closeness and is, therefore, more robust to variations in the embedding scale. Secondly, cosine similarity produces a bounded and interpretable score in [−1, 1], enabling consistent ranking across manuscripts and AEs, whereas negative Euclidean distance is unbounded and lacks a comparable scale. Taken together, these factors make the cosine similarity an appropriate and reliable measure in our AE matching framework.

During feature dimensionality reduction, PCA was employed rather than nonlinear techniques, such as *t*-SNE [7] or uniform manifold approximation and projection (UMAP) [8]. Although *t*-SNE and UMAP can provide strong low-dimensional representations for visualization, their results are highly sensitive to hyperparameter settings and do not offer significant benefits in this study, making them less suitable for downstream similarity computations and large-scale retrieval. PCA, however, offers a deterministic and computationally efficient linear projection that preserves the maximal variance in a stable and reproducible manner, which is important for real-world journal workflows. Furthermore, the reduced feature dimensionality was empirically set to 64, balancing discriminative performance and computational efficiency. Larger dimensions provided an insignificant improvement at a higher computational cost, whereas smaller dimensions led to a noticeable performance degradation in the similarity ranking. Therefore, PCA with 64 dimensions was selected as a good compromise between accuracy, stability, and efficiency.

## Conclusions

In this study, a novel AE matching approach for IEEE TMI is presented that leverages a LLM (ModernBERT) for semantic feature extraction and structured keyword analysis for domain-specific expertise modeling. The system integrates six textual components from each study and applies PCA to compress high-dimensional embeddings and filter semantic noise.

Extensive experiments on historical AE assignment records (internal testing) and AE-authored publications (external testing) have consistently shown that keyword similarity is the primary driver for matching performance, capturing the core topical and methodological alignment between submissions and AEs. Textual similarity alone provides considerably weaker performance; however, it serves as a valuable complementary signal when combined with keywords—particularly after dimensionality reduction using PCA.

Ablation studies on the textual components further revealed that different combinations of titles, abstracts, and keywords yield relatively small performance variations. Structured keywords alone performed the best, followed closely by title and abstract, which contributed the least when used in isolation. This suggests that although textual features offer supplementary information, accurate and comprehensive keyword metadata are the most informative for effective AE-paper matching.

In conclusion, these findings support the proposed practical and interpretable tool for assisting AE assignments that balances efficiency and accuracy. By emphasizing structured keyword matching and enhancing it with optimized textual features, the proposed approach can help reduce editorial workload, improve the precision of AE selection, and facilitate a high-quality peer review process.

## Data Availability

The datasets generated and analyzed during this study are not publicly available due to the internal nature of the IEEE TMI editorial data and the anonymization of AE information to protect confidentiality and privacy. However, the data are available from the corresponding author upon reasonable request.

## Abbreviations

AE: Associate editor
AI: Artificial intelligence
BERT: Bidirectional encoder representations from transformers
LLM: Large language model
PCA: Principal component analysis
TMI: Transactions on Medical Imaging
*t*-SNE: *t*-distributed stochastic neighbor embedding
UMAP: Uniform manifold approximation and projection
